# Euglycemic Diabetic Ketoacidosis in the Setting of Dulaglutide Use

**DOI:** 10.7759/cureus.82143

**Published:** 2025-04-12

**Authors:** Niyati Patel, Anvit Reddy, Kaitlyn N Romero, Pramod Reddy

**Affiliations:** 1 Internal Medicine, University of Florida College of Medicine - Jacksonville, Jacksonville, USA

**Keywords:** dulaglutide, euglycemia diabetic ketoacidosis, glp-1 receptor agonists, type 2 diabetes mellitus, weight loss and obesity

## Abstract

Dulaglutide is a glucagon-like peptide (GLP-1) receptor agonist approved by the Food and Drug Administration in patients with type 2 diabetes mellitus (T2DM) with noted cardiovascular and renal benefits along with weight loss. Dulaglutide's most common adverse effects include fatigue, poor appetite, abdominal pain, nausea, vomiting, and diarrhea. Although typically associated with the use of sodium-glucose cotransporter-2 (SGLT2) inhibitors, there have been case reports of GLP-1 receptor agonists leading to euglycemic diabetic ketoacidosis (DKA). We present a case of dulaglutide use leading to euglycemic DKA, a rare but life-threatening adverse effect after re-initiation of the medication at a higher dose. Our patient presented with non-specific gastrointestinal symptoms and weakness, which both resolved after the initiation of an insulin drip and intravenous fluids.

## Introduction

Dulaglutide is a glucagon-like peptide (GLP-1) receptor agonist that binds to the GLP-1 receptors found on the beta cells in the pancreas. Once bound, serum intracellular cyclic adenosine monophosphate levels rise, leading to the activation of protein kinase A. This then leads to increased insulin secretion and decreased levels of serum glucagon, thereby reducing blood glucose levels. Additionally, GLP-1 receptor agonists lead to delayed gastric emptying by binding to GLP-1 receptors located within the stomach, inhibiting the contraction of gastric muscles, and activating the vagus nerve. GLP-1 receptor agonists are commonly known to cause gastrointestinal side effects, including nausea, vomiting, and diarrhea, which can precipitate dehydration, leading to acute kidney injury, especially in those with known pre-existing kidney disease. They can also lead to pancreatitis, gallbladder issues, vision changes, and thyroid tumors [1].

Rarely, GLP-1 receptor agonists have been seen to cause euglycemic diabetic ketoacidosis (DKA) [2]. However, interestingly enough, with the increased utilization of GLP-1 receptor agonists, the incidence of euglycemic DKA has increased. Euglycemic DKA is a life-threatening emergency that is fatal if unrecognized. Euglycemic DKA is diagnosed when serum blood levels reveal a high anion gap metabolic acidosis along with the presence of serum or urine ketones and a blood glucose level of less than 250 mg/dL. Euglycemic DKA is caused by a state of carbohydrate deficit, usually secondary to inadequate intake, leading to high levels of serum glucagon, epinephrine, and cortisol, along with concomitant low serum insulin [3]. The elevated serum glucagon relative to lower levels of serum insulin leads to ketoacidosis. The metabolic acidosis caused by elevated serum ketones activates compensatory mechanisms of respiratory alkalosis with side effects of nausea, vomiting, and satiety. Euglycemic DKA further leads to glucosuria, which subsequently causes dehydration and increased glucagon secretion. Euglycemic DKA is a known complication seen with the use of sodium-glucose cotransporter-2 (SGLT2) inhibitors, but we present a rare case report of GLP-1 receptor agonists leading to euglycemic DKA.

This article was previously presented as a meeting abstract at the 2024 Southern Regional Meeting on February 13, 2025.

## Case presentation

A 42-year-old woman with a past medical history of uncontrolled type 2 diabetes mellitus (T2DM) and hypertension presented to the emergency room for generalized weakness associated with nausea and vomiting for three days. The patient denied any associated infectious symptoms, recent use of alcohol or illicit drugs, recent surgeries, and previous or current use of metformin or SGLT-2 inhibitors. Of note, the patient endorsed using her first dose of subcutaneous dulaglutide 3 milligrams (mg) three days prior to admission; her symptoms started about one day after starting the medication. She was previously on dulaglutide 1.5 mg weekly a year before this but stopped taking the medication after three months as she was lost to follow-up. When she re-established with her primary care physician, she was restarted on dulaglutide at a dose of 3 mg weekly.

Upon arrival to the emergency room, the patient was found to be afebrile and have a heart rate of 140 beats per minute, respiratory rate of 26 breaths per minute, and blood pressure of 134/104 mmHg. Pertinent laboratory evaluation included a blood glucose level of 162 mg/dL, decreased serum bicarbonate level, potassium within normal limits, increased anion gap, increased beta-hydroxybutyrate, increased lactic acid, and increased hemoglobin A1C (Table 1). Lipase, salicylate, acetaminophen levels, and osmolar gap were all within normal limits. Venous blood gas (VBG) showed a decreased pH with pCO2 and pO2 within normal limits (Table 1). Urinalysis showed elevated ketones but was negative for infectious markers (Table 1). Respiratory panel and urine toxicology were both negative (Table 1). She was admitted to the intensive care unit for euglycemic DKA, where an insulin infusion was initiated at 0.05 units/kg/hour along with intravenous fluids containing dextrose and intravenous potassium when indicated. Once the patient's anion gap was closed twice, the patient was able to tolerate dinner well and was successfully transitioned to subcutaneous insulin. She was discharged on six units of glargine with plans to follow up with her primary care physician; dulaglutide was listed as an allergy for the patient.

**Table 1:** Pertinent admission laboratory results Information in bold helped with the diagnosis of euglycemic DKA: anion gap metabolic acidosis, presence of serum and urine ketones, and blood glucose levels less than 250 mg/dL. DKA: diabetic ketoacidosis, HbA1C: hemoglobin A1C, WBC: white blood cell, BUN: blood urea nitrogen

Admission laboratory testing ordered	Admission laboratory results	Reference ranges
Vbg PH	7.13	7.34-7.36
Vbg CO2	27 mmHg	41-51 mmHg
Vbg O2	49 mmHg	30-40 mmHg
Vbg bicarbonate	9 mmol/L	23-29 mmol/L
K	4.3 mmol/L	3.5-4.5 mmol/L
Serum CO2	9 mmol/L	21-29 mmol/L
Serum glucose	162 mg/dL	71-99 mg/dL
Anion gap	30 mmol/L	4-16 mmol/L
HbA1C	12.3%	4%-6%
Beta-hydroxybutyrate	>46.8 mg/dL	0.2-2.8 mg/dL
Urine ketones	>160 mg/dL	Negative
Urine pregnancy test	Negative	Negative
Urine glucose	Negative	Negative
WBC	8.01 K/μL	4.5-11 K/μL
Lactic acid	2.7 mmol/L	0.7-2 mmol/L
Lipase	34 U/L	0-60 U/L
Acetaminophen	<5 mcg/mL	<35 mcg/mL
Salicylates	<0.3 mg/dL	3-29 mg/dL
Complete respiratory panel	Negative	Negative
Na	139 mmol/L	135-145 mmol/L
Cl	97 mmol/L	98-107 mmol/L
BUN	23 mg/dL	6-22 mg/dL
Creatinine	1.12 mg/dL	0.51-0.96 mg/dL

Table 2 presents the laboratory results of the patient after the initiation of insulin drip and intravenous fluids.

**Table 2:** Laboratory results after initiation of insulin drip and intravenous fluids

Pertinent laboratory testing	Laboratory testing 4 hours after admission	Laboratory testing 8 hours after admission	Laboratory testing 12 hours after admission	Laboratory testing 16 hours after admission	Reference ranges
K	4.5 mmol/L	4.2 mmol/L	3.8 mmol/L	3.8 mmol/L	3.5-4.5 mmol/L
Serum CO2	10 mmol/L	14 mmol/L	20 mmol/L	23 mmol/L	21-29 mmol/L
Serum glucose	131 mg/dL	113 mg/dL	124 mg/dL	101 mg/dL	71-99 mg/dL
Anion gap	29 mmol/L	22 mmol/L	16 mmol/L	13 mmol/L	4-16 mmol/L

## Discussion

The pathophysiology behind GLP-1 agonists leading to euglycemic DKA is thought to be caused by starvation and dehydration that some patients, including our patient, develop after starting the medication. With the most common side effects of GLP-1 agonists being nausea, vomiting, and poor PO intake, the pancreas compensates by increasing glucagon, which leads to lipolysis and ketoacidosis. This formation of ketoacidosis further worsens dehydration, vomiting, and the state of starvation [4]. In comparison, SGLT-2 inhibitors are thought to lead to euglycemic DKA through its mechanism of glucosuria and glucose-induced diuresis. The secretion of glucose in one's urine leads to lower serum glucose levels, for which the body compensates for by decreasing levels of released serum insulin and increasing levels of serum glucagon. Glucagon then activates ketogenesis, increasing the serum ketone levels, which are then reabsorbed by the kidneys rather than excreted. Overall, the state of dehydration due to diuresis, decreased serum insulin levels, and increased serum ketone levels lead to the development of euglycemic DKA.

Literature review revealed that other GLP-1 agonists, other than just dulaglutide, have also been seen to lead to euglycemic DKA, including semaglutide, exenatide, and liraglutide. However, there has been a higher incidence of reported cases in those patients using dulaglutide. Patients have usually reported developing symptoms around 1-3 days after either initiation of the medication or an increase in dosage. Although for several patients, the etiology was secondary to poor carbohydrate intake due to adverse gastrointestinal side effects, some developed euglycemic DKA from concomitant use of SGLT-2 inhibitors or decreased insulin doses due to an increase in GLP-1 agonist dosage.

Euglycemic DKA is a life-threatening emergency that requires prompt diagnosis. It presents with a triad of widened anion gap metabolic acidosis, elevated serum ketones or elevated ketones in urine, and normal serum glucose levels usually below 250 mg/dL [5]. Common risk factors include dehydration, pregnancy, prolonged fasting, pancreatitis, abuse of cocaine, infections, bariatric surgery, liver cirrhosis, gastroparesis, insulin pump malfunction, and glycogen storage disorders [6]. The most common medication class to cause this is SGLT-2 inhibitors, although the incidence of GLP-1 agonists causing this is rising [7]. In our patient, all other possible etiologies of euglycemic DKA were excluded, including the concomitant use of SGLT-2 inhibitors, pointing to dulaglutide as the culprit [8]. Starvation ketosis was able to be ruled out in this patient given the patient's history of recent re-initiation of a GLP-1 agonist as well as her symptoms of abdominal pain, nausea, and vomiting. Patients with starvation ketosis usually present with fatigue and weight loss as opposed to acute gastrointestinal symptoms. The laboratory findings of starvation ketosis and euglycemic DKA can present similarly, and for this reason, a patient's symptoms and history are essential in distinguishing the two.

Euglycemic DKA is difficult to diagnose when compared to DKA as it is not accompanied by hyperglycemia. Euglycemic DKA should be promptly treated with isotonic fluids such as lactated ringers with the addition of dextrose 5% or 10% if hypoglycemia persists, an insulin drip at the rate of 0.1 units/kg/hour or 0.05 units/kg/hour, and supplemental intravenous potassium. As with DKA, serum glucose levels should be checked hourly, and electrolytes, especially potassium, should be monitored every four hours. Euglycemic DKA and DKA are treated similarly as they both require prompt initiation of intravenous fluids, an insulin drip, and repletion of electrolytes as indicated. However, euglycemic DKA often requires intravenous dextrose along with intravenous fluids, given the euglycemic state and the risk of hypoglycemia while the patient is still requiring an insulin drip. Treatment should be continued until the anion gap closes and metabolic acidosis resolves [9]. If euglycemic DKA is left untreated, it can lead to hypovolemic shock, respiratory demise, cerebral edema, seizures, myocardial infarction, or death [10].

Determining the etiology of euglycemic DKA and educating patients on the trigger is essential in preventing recurrence. As in our patient, GLP-1 agonists were listed as an allergy to ensure that this patient was not prescribed this type of medication again.

## Conclusions

GLP-1 agonists, including dulaglutide, are becoming more widely prescribed than just for diabetes due to their significant benefits in obesity, cardiovascular, and renal health. This case report aims to increase awareness of the GLP-1 agonist's potential to lead to life-threatening euglycemic DKA. Prior to prescribing this medication, physicians should educate patients on the possible adverse reactions and symptoms of euglycemic DKA so they can seek medical attention promptly. Clinicians should maintain a high degree of suspicion for euglycemic DKA when patients present with non-specific gastrointestinal symptoms, especially after recent initiation or up-titration of GLP-1 agonists. Prompt diagnosis and treatment with intravenous fluids and insulin drip can be life-saving.
